# Advanced Structural Concrete Materials in Bridges

**DOI:** 10.3390/ma15238346

**Published:** 2022-11-24

**Authors:** Eva Olivia Leontien Lantsoght

**Affiliations:** 1 Concrete Structures, Department of Engineering Structures, Civil Engineering and Geosciences, Delft University of Technology, 2628 CN Delft, The Netherlands; e.o.l.lantsoght@tudelft.nl; 2 Colegio de Ciencias e Ingenierías—Politécnico, Universidad San Francisco de Quito, Quito EC170901, Ecuador

Many existing and newly constructed bridges are made of reinforced and prestressed concrete. Advanced concrete materials play an increasingly important role in concrete bridges, facilitating the strengthening and repair of existing bridges, fast replacement solutions for parts of existing bridges, and for the design of novel challenging bridge projects. The development of advanced concrete materials and their structural applications is, thus, an important topic in the built environment.

The articles presented in this Special Issue bring together research insights, practical applications, and discussions on how to develop better structural concrete materials for bridge engineering practices. Since articles were written both by materials scientists and bridge engineers, a wide view on the topic is presented.

One example of an advanced structural concrete material is ultra-high-performance fiber-reinforced concrete (UHPFRC). With research on this topic having reached a high level of maturity, practical applications of this material are becoming increasingly more common. A key practical issue is related to the resulting fiber content, fiber orientation, and efficiency factor of the structural elements, particularly relevant for thin elements. This property determines the post-cracking tensile strength of the UHPFRC. In [1], the authors propose a nondestructive evaluation (NDE) method for the quality control of UHPFRC in industrial settings and provide recommendations for the efficient implementation of this method. In [2], the authors study the interface shear strength of UHPFRC by testing Z-shaped specimens. They found that the steel fiber type had little effect on the shear strength and ductility, while increasing the length of the steel fibers improved the ductility and slightly reduced the shear strength. Ultimately, the authors proposed an interaction formula for the shear and compressive strength to predict the shear capacity of cast-in-place UHPFRC structures.

Advances in steel-fiber-reinforced concrete include the development of steel-fiber-reinforced expanded-shale lightweight concrete (SFRELC) [3]. In particular, the authors explored the fatigue strength of composite SFRELC-RC beams through experiments. The results showed that with the increase in SFRELC depth and the volume fraction of steel fibers, the fatigue life of the test beams was prolonged. The outcome of the research was a method for predicting the stress level, the stress amplitude of the longitudinal tensile rebar, and the degenerated flexural stiffness of SFRELC superposed beams with a fatigue life. These insights are important for the development of composite SFRELC-RC bridge elements, making optimal use of both materials.

Another example of an advanced structural concrete material is concrete with an MgO expansive agent and steel fibers added to the mix. This improved concrete mix [4] was applied in the construction of the Xiaoqing River Bridge to provide protection against shrinkage cracking. Measurements on the deck after casting showed that the MgO expansive agent could effectively prevent shrinkage cracks. The reinforcement ensured that the MgO expansive agent did not further expand in time, and adding steel fibers resulted in a three-dimensional restraint of further MgO expansion. The proposed improved mix not only counteracted shrinkage cracking, but also improved the mechanical properties of the bridge deck.

For the prefabrication industry, a better understanding of the structural behavior can result in the optimization of the resulting prefabricated elements. A topic of particular interest is the interface bond strength and anchorage performance of the steel reinforcement (ribbed and plain bars) in the prefabricated concrete. The experimental study of [5] resulted in a proposal for the anchorage length in prefabricated elements, which could be applied to prefabricated bridge elements. An application of precast, prestressed concrete deck slabs with high-performance concrete (HPC) is given in [6]. The developed deck slabs had a smaller cross-section, and, thus, reduced weight, as well as better durability than traditional solutions. However, the joint between the slab panels was considered a weak spot in the deck. Therefore, the authors experimentally evaluated slabs with joints. The experiments showed that the deck slabs fulfilled all the static and fatigue performance requirements, as well as serviceability requirements. This solution could, thus, be applied in bridges.

For existing bridges, the first challenge is often the proper assessment of the structure; the information is important for the evaluation of various possible solutions, such as for the strengthening of the structure. In [7], the authors determine the durability and strength of a 95-year-old concrete built-in arch bridge based on the mechanical, physical, and chemical properties of the concrete. The insights of these experiments, as well as the literature review carried out by the authors, provide guidance to bridge engineers who are faced with the problem of assessing existing bridges using historical concrete mixes. Then, the authors used this information in [8] to perform a structural analysis of the bridge. Using insights from the material investigation resulted in the conclusion that the bridge fulfilled the code requirements and that it could remain in service. Such evaluations are of the utmost importance before determining whether a replacement or the application of a strengthening technique to existing bridges would be needed.

On the topic of strengthening existing bridges, various advanced structural concrete materials are proposed. Experiments on reinforced concrete beams strengthened with textile-reinforced concrete (TRC) and fiber–textile-reinforced concrete (F/TRC) showed the beneficial effects of this strengthening technique at the ultimate limit state, as well as in the serviceability limit state [9]. A second paper [10] also addresses the use of TRC for strengthening, and, in particular, for bridge deck slabs. The proposed strengthening solution, SMART-DECK, consists of a carbon-fiber-reinforced polymer reinforcement together with a high-performance mortar. The experiments addressed both the flexural and shear strength of the TRC-strengthened slab segments, and the authors found a high increase in the bending and shear capacity when using the proposed strengthening approach.

UHPFRC is also proposed for the strengthening of existing bridges [11]. Experiments have shown the applicability of UHPFRC for strengthening, but a key issue that remains is the estimation of the bond strength to the existing concrete, especially for numerical applications. In this paper, the authors experimentally and numerically investigated this bond strength as a function of the roughening of the existing concrete surface. From this research, the authors proposed to use a zero-thickness volume model in nonlinear finite element analyses to properly model the bond properties.

Polyvinyl alcohol fiber-reinforced engineering cementitious composites (PVA-ECCs) are proposed in another paper [12], where the authors address the challenge of closing and repairing a portion of a bridge while the other portion is left open to traffic. Consequently, newly placed PVA-ECC bridge repairs (NP-ECC-BRs) are exposed to continuous traffic vibrations (TRVs), even during the setting periods. Therefore, they address the TRVs and their influence on the flexural properties of the PVA-ECC repairs. The authors found that, indeed, the flexural capacity decreased when the PVA-ECC repairs were subjected to TRVs, and that the flexural deformation capacity was not affected.

Another novel method for strengthening was proposed in [13], in which carbon-fiber-reinforced polymer (CFRP) was applied as a near-surface-mounted reinforcement (NSMR). The resulting system had a high ductility and, thus, an advantage over typical adhesively-bonded CFRP systems that can result in a brittle concrete delamination failure, reduced warning, and the consequent inefficient use of the CFRP. Thanks to the high ductility of the CFRP NSMR system, a high utilization of the CFRP can be reached as well. The proposed strengthening technique was studied experimentally, showing promising results.

Ultimately, [14] proposes a conceptual approach for the replacement of existing bridges. As high numbers of bridges are reaching the end of their originally devised service life, smart concepts for this replacement task are needed. The authors propose a fast and hindrance-free method for their replacement. A combination of recent innovations in construction technology, such as advanced cementitious materials (ACMs), structural health monitoring (SHM) techniques, advanced design methods (ADMs), and accelerated bridge construction (ABC), is being used. Thus, the authors show how all recent research insights can be combined to tackle this major societal challenge at hand in various countries around the world.

These presented papers provide insight into the current state-of-the-art of advanced structural concrete materials in bridge applications, and show the way towards the practical application of these materials. I appreciate all author contributions and sincerely value the time and effort spent in preparing these articles. I would also like to thank all reviewers who contributed to this Special Issue for their time, effort, and valuable remarks to the articles. Finally, I would like to thank the academic editors of *Materials,* who helped handle the manuscripts of this Special Issue, as well as the journal staff, who provided expert assistance to the development of this Special Issue at every step of the way.
